# Fecal microbiota transplantation mitigates lipopolysaccharide-induced oxidative stress in weaned piglets by modulating gut microbiota and enhancing riboflavin metabolism

**DOI:** 10.1186/s40104-025-01330-6

**Published:** 2026-01-16

**Authors:** Jixiang Ma, Mengqi Liu, Junying Xu, Boshuai Liu, Yalei Cui, Yinghua Shi

**Affiliations:** 1 College of Animal Science and Technology, Henan Agricultural University, Zhengzhou, 450002 China; 2 Henan Key Laboratory of Innovation and Utilization of Grassland Resources, Zhengzhou, 450002 China; 3 Henan Forage Engineering Technology Research Center, Zhengzhou, Henan 450002 China

**Keywords:** Fecal microbiota transplantation, Flavin adenine dinucleotide, Lipopolysaccharide, Oxidative stress, Short-chain fatty acids, Weaned piglets

## Abstract

**Background:**

During the weaning phase, piglets are exposed to significant physiological and environmental stressors, which disrupt the balance of their intestinal microbiota and often lead to severe diarrhea. Previous studies have demonstrated that alfalfa fiber, derived from the stems and leaves of alfalfa, can effectively alleviate diarrhea in piglets. Additionally, multiple studies have highlighted the potential of fecal microbiota transplantation (FMT) in mitigating diarrhea in various models of intestinal diseases in young animals. However, the specific mechanisms by which FMT from targeted sources alleviates diarrhea in weaned piglets remain to be fully elucidated.

**Results:**

In this study, FMT from donor piglets fed an alfalfa fiber-supplemented diet effectively alleviated diarrhea, improved intestinal morphology, and enhanced gut barrier function in weaned piglets. FMT further promoted the colonization of beneficial bacterial genera (including *UCG-005*, *unclassified Lachnospiraceae*, *Lachnospiraceae AC2044 group*, *UCG-002*, *Candidatus Saccharimonas*, and *Lachnospiraceae ND3007 group*) while inhibiting the detrimental genus *Tyzzerella*, consequently enhancing the production of short-chain fatty acids (SCFAs). Additionally, FMT upregulated riboflavin metabolism, leading to elevated flavin adenine dinucleotide (FAD) levels and increased glutathione reductase activity, thereby collectively attenuating lipopolysaccharide (LPS)-induced oxidative stress and contributing to intestinal health.

**Conclusions:**

We found that FMT modulates the structure of the gut microbiota, enhances microbial diversity and composition, increases the production of SCFAs, and upregulates riboflavin metabolism to elevate FAD levels. These changes collectively enhance immune and antioxidant capacities, thereby alleviating diarrhea.

**Supplementary Information:**

The online version contains supplementary material available at 10.1186/s40104-025-01330-6.

## Introduction

The weaning phase is a critical period that significantly impacts the growth and development of piglets. During this stage, piglets face physiological challenges such as an underdeveloped gastrointestinal tract, an immature intestinal microbiota structure, and the transition from maternal milk to solid feed [1]. Furthermore, external environmental changes and social stressors exacerbate gastrointestinal dysfunction, triggering intestinal inflammation and leading to diarrhea [2]. In fact, the secretion of enterotoxins by potential pathogenic bacteria in the gut is a major contributing factor to diarrhea in piglets [3]. Severe diarrhea not only hinders the healthy development of piglets and reduces their growth performance but also results in substantial economic losses for the swine industry [4]. The gut microbiota has been recognized as an essential component of intestinal architecture, playing a pivotal role in training host immunity, regulating intestinal endocrine functions, and driving host metabolism [5]. A stable gut microbiota can mitigate the damage to the intestinal barrier caused by potentially harmful pathogens, prevent the invasion of toxic substances such as lipopolysaccharides (LPS) into the bloodstream, enhance intestinal immunity, and maintain intestinal homeostasis [6–8]. Furthermore, certain gut bacteria such as *Bacteroides* spp. and *Ruminococcus* spp. are efficient producers of acetate and propionate [9], while members of the Lachnospiraceae family can produce acetate and also generate butyrate [10]. It has been reported that short-chain fatty acids (SCFAs) derived from the gut microbiota can regulate intestinal barrier function and host immunity through multiple pathways [11, 12]. In addition, the gut microbiota produces metabolites such as bile acids and choline, critical for the maintenance of intestinal barrier function and host health [13].

Dietary fiber promotes gastrointestinal motility in the host, reduces intestinal infections, and inhibits pathogen adhesion to the gastrointestinal mucosa [14]. On the other hand, dietary fiber effectively modulates the structure of the gut microbial community, facilitates the colonization of beneficial bacteria, reduces potential pathogens and health-threatening metabolites, and maintains intestinal homeostasis [15, 16]. *Medicago sativa* (alfalfa), renowned as the "king of forage", is rich in nutrients and dietary fiber [17]. Early studies reported that alfalfa meal regulates gut microbiota structure, improves intestinal health, and alleviates diarrhea in piglets [18]. High fiber content alfalfa fiber can be obtained by crushing the stalks after screening and separating the alfalfa stalks and leaves. Studies have shown that supplementing piglet diets with alfalfa fiber enhances growth performance and mitigates diarrhea [19]. Furthermore, dietary inclusion of alfalfa fiber alleviates LPS-induced dysbiosis of gut microbiota, reduces intestinal inflammation, and improves intestinal health in piglets [20]. Fecal microbiota transplantation (FMT) has long been utilized to remodel gut microbiota in diseased hosts, serving as an effective therapeutic strategy for treating intestinal disorders [21, 22]. Recent studies demonstrate that early-life FMT from diverse sources enhances gut microbial composition, intestinal mucosal immunity, and developmental outcomes in piglets, thereby improving growth performance [23–26]. These findings suggest that FMT may serve as a strategic intervention to alleviate weaning stress in weaned piglets.

Based on previous findings that dietary supplementation with alfalfa fiber alleviates diarrhea and enhances growth performance in weaned piglets [20], this study further explores the effects of FMT on the growth performance of weaned piglets and its regulatory mechanisms. In this study, we investigated how fecal microbiota from donor piglets fed alfalfa fiber influenced the gut microbiota and metabolic profiles of weaned piglets challenged with an LPS-induced oxidative stress model to understand the physiological regulatory roles of FMT and reveal its effects on modulating the gut microbiota and metabolic processes in weaned piglets.

## Materials and Methods

### Preparation of fecal microbiota suspension

In this study, six healthy 60-day-old piglets fed a basal diet supplemented with 2.5% alfalfa fiber were selected as fecal donors. The donor piglets had no recent history of gastrointestinal diseases or antibiotic treatments. The preparation of the fecal microbiota suspension was performed following the method described by Hu et al. [27]. Briefly, fresh fecal samples from donor pigs were immediately diluted (1:5, w/v) in sterile saline buffer supplemented with 10% glycerol. The diluted samples were then homogenized using a 0.25-mm filter and transferred into sterile cryotubes for storage at −80 °C until further use. Detailed information on the donor piglets is provided in Table S1.

### FMT experiment

The FMT experiment consisted of two trials (Trial 1 and Trial 2). In trial 1, a total of 36 early-weaned castrated male (Duroc × Landrace × Yorkshire) piglets, aged 28 d with an initial body weight of 8.65 ± 0.70 kg, were randomly assigned to two treatment groups: NS group (Control group, received sterile saline buffer) and NF group (FMT group, received fecal microbiota suspension). Each treatment group consisted of six replicates, with three piglets per replicate. The adaptation period lasted 3 d, followed by a 14-d experimental period. During the experimental period, both groups were fed the basal diet, and piglets received an oral gavage of 2 mL of either sterile saline buffer or fecal microbiota suspension every 2 d for a total of seven administrations. At the end of the experiment, piglets were fasted for 12 h, and one piglet per replicate was randomly selected for blood sample collection via the anterior vena cava. Subsequently, piglets were anesthetized with pentobarbital sodium (40 mg/kg BW). Once no signs of life were detected, the jugular vein was severed using a scalpel to ensure complete exsanguination. Finally, the abdominal cavity was opened, and intestinal tissue samples were collected. In trial 2, a total of 54 early-weaned castrated male (Duroc × Landrace × Yorkshire) piglets, aged 28 d with an initial body weight of 8.50 ± 0.65 kg, were randomly assigned to three treatment groups: NS group (Received oral gavage of fecal microbiota dilution buffer + intraperitoneal injection of sterile saline), NL group (Received oral gavage of fecal microbiota dilution buffer + intraperitoneal injection of LPS) and FL group (FMT + intraperitoneal injection of LPS). Each treatment group consisted of six replicates, with three piglets per replicate. The adaptation period lasted 3 d, followed by a 14-d experimental period. During the experimental period, piglets received oral gavage of 2 mL of either fecal microbiota dilution buffer or fecal microbiota suspension every 2 d for a total of seven administrations. On the final day of the experiment, piglets in the NS, NL, and FL groups received an intraperitoneal injection of either LPS (100 μg/kg BW, *Escherichia coli* O55:B5, Sigma Chemical, USA) or sterile saline. After 4 h, one piglet per replicate was randomly selected, and blood, intestinal tissue, and gut content samples were collected.

Prior to the start of the trial, the pig housing facilities underwent strict disinfection, and the indoor temperature was maintained at 24 to 26 °C, with a 12 h light/dark cycle. Throughout the FMT experiment, all piglets had ad libitum access to feed and water. Body weight was recorded before and after the experiment following a 12-h fasting period, and daily feed intake was monitored.

### Growth performance and diarrhea rate

During the experimental period, the same researcher recorded the number of piglets experiencing diarrhea in each replicate daily at 17:00. Diarrhea was defined based on unformed feces and anal redness, and the diarrhea rate was calculated accordingly. Based on initial body weight (IBW), final body weight (FBW), and daily feed intake, the following growth performance parameters were calculated:

- Diarrhea rate = (Total number of diarrhea occurrences per replicate/Total number of pigs in the experiment/Total experimental days) × 100%
- ADG = (FBW − IBW)/Total experimental days.
- ADFI = Total feed intake during the experiment/Total experimental days.

### Biochemical analysis

The levels of interleukin-1β (IL-1β), interleukin-10 (IL-10), interleukin-22 (IL-22), tumor necrosis factor-α (TNF-α), immunoglobulin A (IgA), immunoglobulin G (IgG), immunoglobulin M (IgM), heat shock protein 70 (HSP70), diamine oxidase (DAO), reduced glutathione (GSH), oxidized glutathione (GSSG), and glutathione reductase (GR) were measured according to the instructions provided in the ELISA kits (Shanghai Enzyme-linked Biotechnology Co., Ltd., China). The levels of total superoxide dismutase (T-SOD), total antioxidant capacity (T-AOC), malondialdehyde (MDA), and reactive oxygen species (ROS), as well as protein quantification in cells and tissues using the BCA method, were determined following the protocols provided in the assay kits (Nanjing Jiancheng Bioengineering Institute, China). Each sample was analyzed with a minimum of three technical replicates to ensure measurement precision.

### Intestinal morphology

Under the premise of preserving intestinal morphology, approximately 2-cm segments of the duodenum, jejunum, ileum, and colon were carefully dissected. The intestinal contents were gently rinsed with PBS buffer, and the tissues were fixed in 4% paraformaldehyde solution. The tissues were then processed into paraffin-embedded sections following the standard histological embedding procedure for subsequent morphological analysis. The sections were examined and photographed under an optical microscope (Motic, China). The villus height (VH) and crypt depth (CD) were measured, and the villus-to-crypt ratio (VCR) was calculated.

### Immunofluorescence

The distribution of colonic barrier proteins zonula occludens-1 (ZO-1) and Occludin in weaned piglets were examined by immunofluorescence, following the method described by Liu et al. [28]. Briefly, colonic tissue sections were incubated with primary antibodies against ZO-1 and Occludin overnight at 4 °C. Subsequently, horseradish peroxidase (HRP)-conjugated IgG secondary antibodies were applied. The sections were then incubated with DAPI for 10 min in the dark. The samples were examined using a Nikon Eclipse C1 microscope (Nikon, Japan) and imaged with a Nikon DS-U3 imaging system (Nikon, Japan). Fluorescence intensity was quantified using ImageJ software.

### PAS staining

Periodic Acid-Schiff (PAS) staining was performed to identify goblet cells in the colonic tissue of piglets. Briefly, paraffin-embedded colonic tissue sections were deparaffinized to water and stained with PAS staining solution. The sections were subsequently washed thoroughly with running water multiple times. After dehydration and mounting with a coverslip, the stained sections were examined under a Nikon Eclipse E100 microscope (Nikon, Japan), and images were captured using a Nikon DS-U3 imaging system (Nikon, Japan).

### Real-time quantitative PCR

Total RNA was extracted from piglet colonic tissues using the TRIzol method according to the manufacturer's protocol (Invitrogen, USA). RNA concentration was determined using a Nanodrop 2000 microvolume spectrophotometer (Thermo, USA). cDNA synthesis was performed following the protocol provided by the reverse transcription kit (Toyobo, Japan). Gene-specific primers were designed based on sequences retrieved from the NCBI database, with *GAPDH* serving as the reference gene. Primer sequences and parameters are detailed in Table S2. mRNA expression levels were quantified by qPCR using the ChamQ Universal SYBR qPCR Master Mix (Vazyme, Nanjing, China) on a LightCycler 96 Real-time PCR system (Roche, Switzerland). The relative mRNA expression levels of each gene were calculated using the 2^−ΔΔCT^ method as described by Livak et al. [29]. Each sample was analyzed with a minimum of three technical replicates to ensure measurement precision.

### 16S rRNA gene high-throughput sequencing

Four colonic content samples were randomly selected from each treatment group. Total bacterial DNA from the colonic contents was extracted using the E.Z.N.A. DNA Stool Mini Kit (Omega, USA). After DNA extraction, genomic DNA quality was assessed using 1% agarose gel electrophoresis. The V3–V4 variable regions of the 16S rRNA gene were amplified by PCR using the primers 338F (5'-ACTCCTACGGGAGGCAGCAG-3') and 806R (5'-GGACTACHVGGGTWTCTAAT-3'). The PCR products were quantified and assessed using a Quantus™ Fluorometer (Promega, USA). A PE amplicon library was constructed and sequenced on the Illumina MiSeq PE300 platform (Shanghai Meiji Biomedical Technology Co., Ltd., China). Raw sequencing data were quality-controlled using fastp (https://github.com/OpenGene/fastp, version 0.20.0). Sequences were clustered into OTUs based on 97% similarity, and chimeric sequences were removed. Species classification and annotation of each sequence were performed using the RDP classifier (http://rdp.cme.msu.edu/, version 2.2). Alpha diversity analysis (including Sobs, Chao, and ACE indices), Principal Coordinates Analysis (PCoA), LEfSe analysis, and differential genus analysis of colonic microbiota were performed using the Majorbio Cloud Platform (www.majorbio.com) and OmicStudio Cloud Platform (www.omicstudio.cn). The Spearman correlation analysis between differential colonic genera and serum biochemical factors was conducted on the BioinCloud Platform (www.bioincloud.tech). The significance threshold for Kruskal–Wallis H test was set at *P* < 0.05, and LDA ≥ 3 was used to identify biomarkers among different treatment groups. The raw sequencing data have been uploaded to the NCBI database (BioProject: PRJNA1181642).

### Determination of SCFAs

Colonic contents from piglets were weighed and dissolved in deionized water at a ratio of 2 mL per 0.1 g of sample. After sonication for 30 min, the mixture was centrifuged at 9,500 × *g* for 10 min. A 1 mL aliquot of the supernatant was transferred to a 2-mL centrifuge tube and mixed with 0.2 mL of 25% metaphosphoric acid solution. The sample was incubated at 4 °C for 30 min and then centrifuged again at 9,500 × *g* for 10 min. A 100 µL aliquot of the supernatant was transferred to a 1.5-mL centrifuge tube and diluted tenfold with deionized water (by adding 900 µL of deionized water). The diluted sample was filtered through a 0.22-µm membrane filter and analyzed for SCFA concentrations using a gas chromatography system (Agilent 6890N, Palo Alto, CA, USA).

### LC–MS untargeted metabolomics analysis

Metabolomics analysis based on liquid chromatography-mass spectrometry (LC–MS) was performed by Majorbio (Shanghai, China). The main steps were as follows: 50 mg of piglet colonic contents were placed in a centrifuge tube, to which grinding beads and a metabolite extraction solution (320 μL methanol + 80 μL water + 0.008 mg L-2-chlorophenylalanine) were added. The mixture was then ground at −10 °C at 50 Hz for 6 min, followed by sonication at 5 °C and 40 kHz for 30 min. After allowing the samples to stand at −20 °C for 30 min, they were centrifuged at 13,000 × *g* for 15 min at 4 °C. The supernatant was transferred into LC autosampler vials. Additionally, aliquots of the supernatant from each sample were pooled to generate a quality control sample. LC–MS analysis was conducted using an ultra-high performance liquid chromatography tandem Fourier transform mass spectrometry system (Thermo Fisher Scientific, USA). The raw LC–MS data were processed with Progenesis QI (Waters Corporation, Milford, USA), and the mass spectral information was matched to databases to obtain metabolite profiles. All metabolite data were uploaded to the Majorbio Cloud Platform (www.majorbio.com) for further analysis, and principal component analysis (PCA) was performed on the Gene Cloud Platform (www.genescloud.cn). Differential metabolites were identified based on the variable importance in projection (VIP) values obtained from an OPLS-DA model and Student’s *t*-test *P*-values, with metabolites having VIP > 1 and *P* < 0.05 being considered as significant.

### Caco-2 cell experiment

Flavin adenine dinucleotide disodium (FAD, purity ≥ 95%) was purchased from Macklin (Shanghai, China) and diluted in PBS buffer. Human cloned colon adenocarcinoma Caco-2 cells (Beijing University of Chinese Medicine, China) were cultured in DMEM (GIBCO, USA) supplemented with 10% fetal bovine serum (FBS, Biological Industries, Israel) at 37 °C with 5% carbon dioxide. Caco-2 cells were treated with different concentrations (0, 0.1, 0.5, 1.0, 5.0, 10 μg/mL) of LPS to induce oxidative stress. Cell viability was detected using a CCK8 kit (Beyotime, Shanghai, China), and T-AOC and ROS levels were measured to determine the optimal LPS concentration for inducing oxidative stress. Additionally, Caco-2 cells were pretreated with different concentrations (0, 0.1, 0.5, 1.0, 5.0, 10, 50 μmol/L) of FAD and then induced with the optimal LPS concentration for 24 h. Cell viability and antioxidant levels were measured to determine the optimal FAD concentration. Finally, the cell assay was divided into four groups: CON group (no addition of FAD or LPS), LPS group (5.0 μg/mL LPS), FAD group (50 μmol/L FAD), and FAD + LPS group (50 μmol/L FAD + 5.0 μg/mL LPS). The treatment time for the cells was 24 h, and glutathione and antioxidant capacity indicators were measured. The ROS expression level in cells was detected using the fluorescent probe DCFH-DA (Beyotime, Shanghai, China). The cells were scanned and photographed under a Caseviewer (Pannoramic MIDI, 3DHISTECH) scanner, and the fluorescence intensity was quantified using ImageJ software.

### Statistical analysis

Except for the analysis of 16S rRNA and LC–MS untargeted metabolomics, data from two groups were analyzed using Student's *t*-test with SPSS 20.0, while data from multiple groups were analyzed using one-way analysis of variance (ANOVA). Duncan's method was used for multiple comparisons among the groups, with a significance level of *P* < 0.05.

## Results

### FMT alleviates diarrhea in weaned piglets

**Fig. 1:**
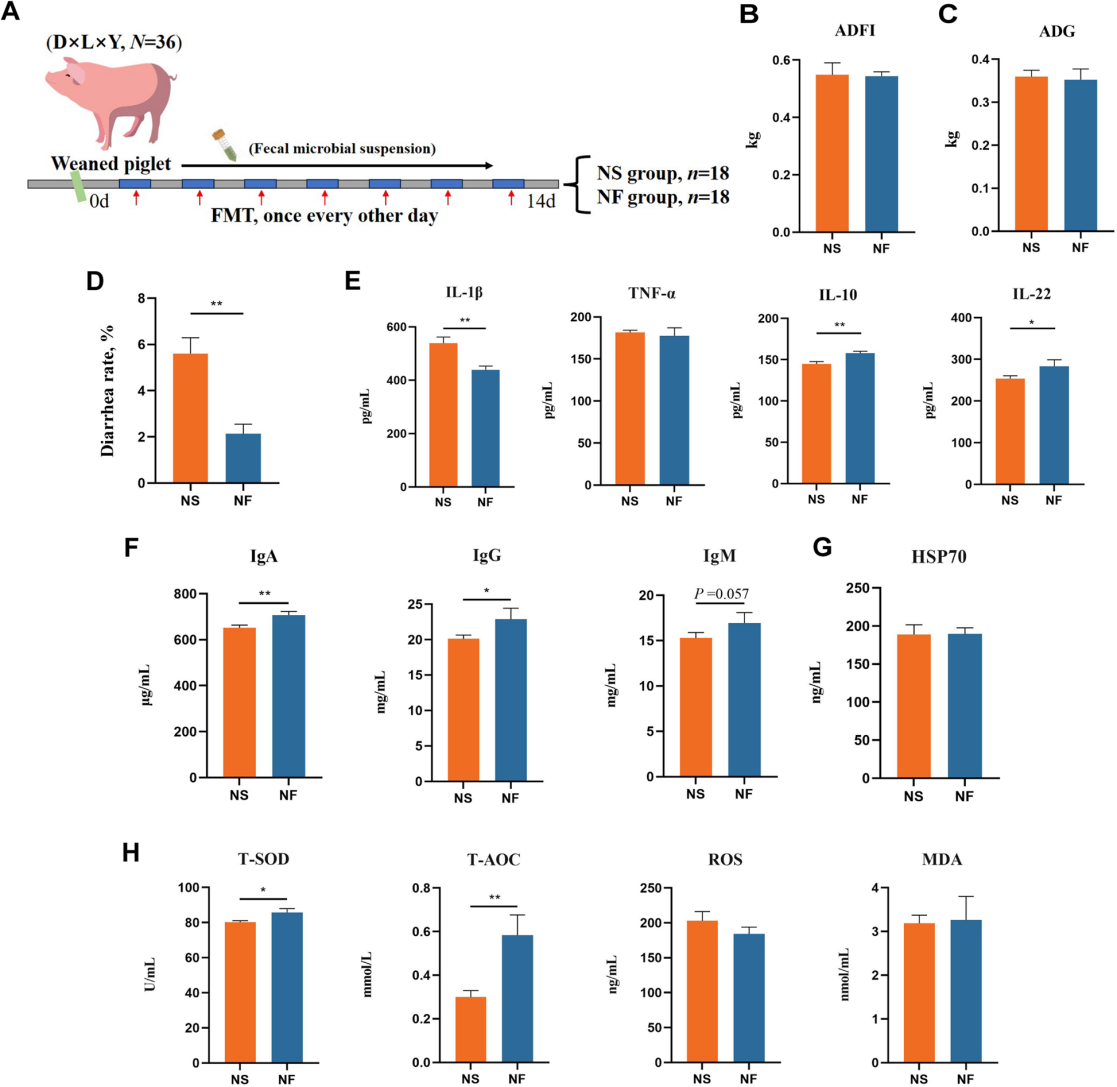
FMT alleviates diarrhea in weaned piglets. **A** Schematic diagram of the trial 1 model. **B** Average daily feed intake. **C** Average daily gain. **D** Diarrhea rate. **E** Effects of FMT on serum inflammatory factors (IL-1β, TNF-α, IL-10, and IL-22) in piglets. **F** Effects of FMT on serum immunoglobulins (IgA, IgG, and IgM) in piglets. **G** Effects of FMT on serum HSP70 levels in piglets. **H** Effects of FMT on serum antioxidant capacity (T-SOD, T-AOC, ROS, and MDA) in piglets. ^*^P < 0.05, ^**^P < 0.01. Data are shown as mean ± SD. *n* = 6

Based on the favorable effects of alfalfa fiber meal diet in alleviating diarrhea in weaned piglets [20], we speculate that this effect is mediated through the gut microbiota. To test this, we prepared fecal microbiota suspensions from donor piglets fed the alfalfa fiber meal diet and transferred them to recipient piglets to assess the effects (Fig. 1A). Our results revealed that FMT significantly alleviated the diarrhea rate in weaned piglets (Fig. 1B–D). Additionally, FMT significantly reduced the serum concentration of the inflammatory cytokine IL-1β and significantly increased the concentrations of IL-10 and IL-22 (Fig. 1E). Meanwhile, FMT significantly increased the serum concentrations of immunoglobulins IgA and IgG, as well as the activity of T-SOD and the concentration of T-AOC (Fig. 1F–H).

### FMT improves intestinal health in weaned piglets

**Fig. 2:**
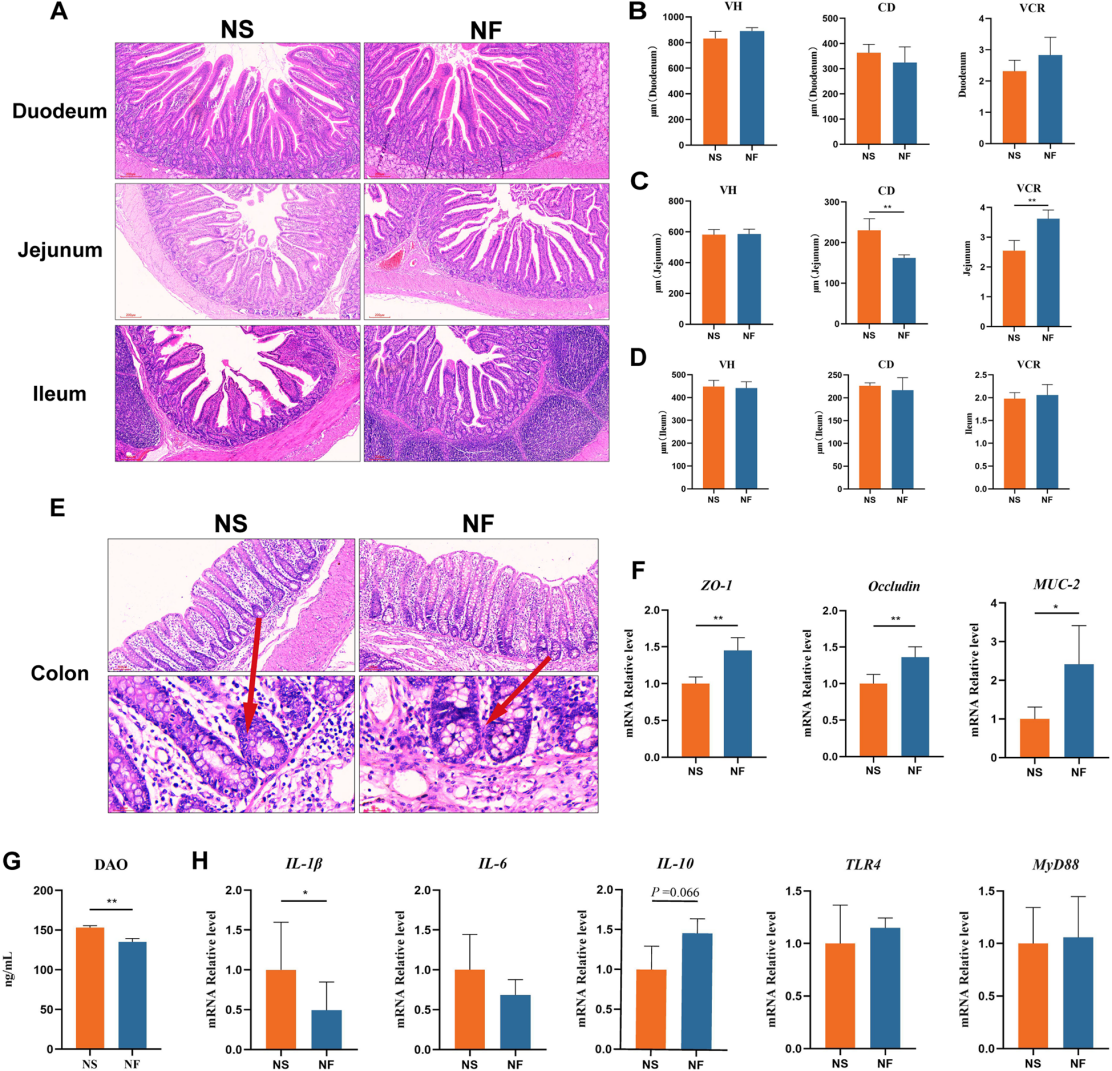
FMT improves intestinal health in weaned piglets. **A** Effects of FMT on small intestinal morphology in piglets. **B** Effects of FMT on duodenal VH, CD, and VCR in piglets. **C** Effects of FMT on jejunal VH, CD, and VCR in piglets. **D** Effects of FMT on ileal VH, CD, and VCR in piglets. **E** Effects of FMT on colonic development in piglets, with red arrows indicating crypt development. **F** Effects of FMT on the gene expression of colonic barrier proteins (*ZO-1*, *Occludin*, and *MUC-2*) in piglets. **G** Effects of FMT on serum permeability marker DAO in piglets. **H** Effects of FMT on colonic inflammation and NF-κB pathway factors (*IL-1β*, *IL-6*, *IL-10*, *TLR4*, and *MyD88*) in piglets. ^*^P < 0.05, ^**^P < 0.01, Data are shown as mean ± SD. *n* = 6

A favorable intestinal morphological structure facilitates nutrient absorption by the host, resists the penetration of toxic and harmful substances into the bloodstream, and maintains body health [30]. We found that FMT significantly reduced jejunal CD and increased the jejunal VCR in weaned piglets (Fig. 2A–D). Additionally, FMT improved colonic crypt development and significantly increased the gene expression levels of the *ZO-1* and *Occludin*, as well as the *MUC-2*, while decreasing serum DAO concentration (Fig. 2E–G). Meanwhile, we also found that the expression of *IL-1β* gene in the colon of weaned piglets was significantly reduced and the expression of *IL-10* gene tended to be elevated after FMT treatment (Fig. 2H). These results indicated that FMT improved intestinal morphology and development, reduced intestinal inflammation level, and enhanced intestinal barrier function in piglets.

### FMT alleviates LPS-induced oxidative stress in weaned piglets

**Fig. 3:**
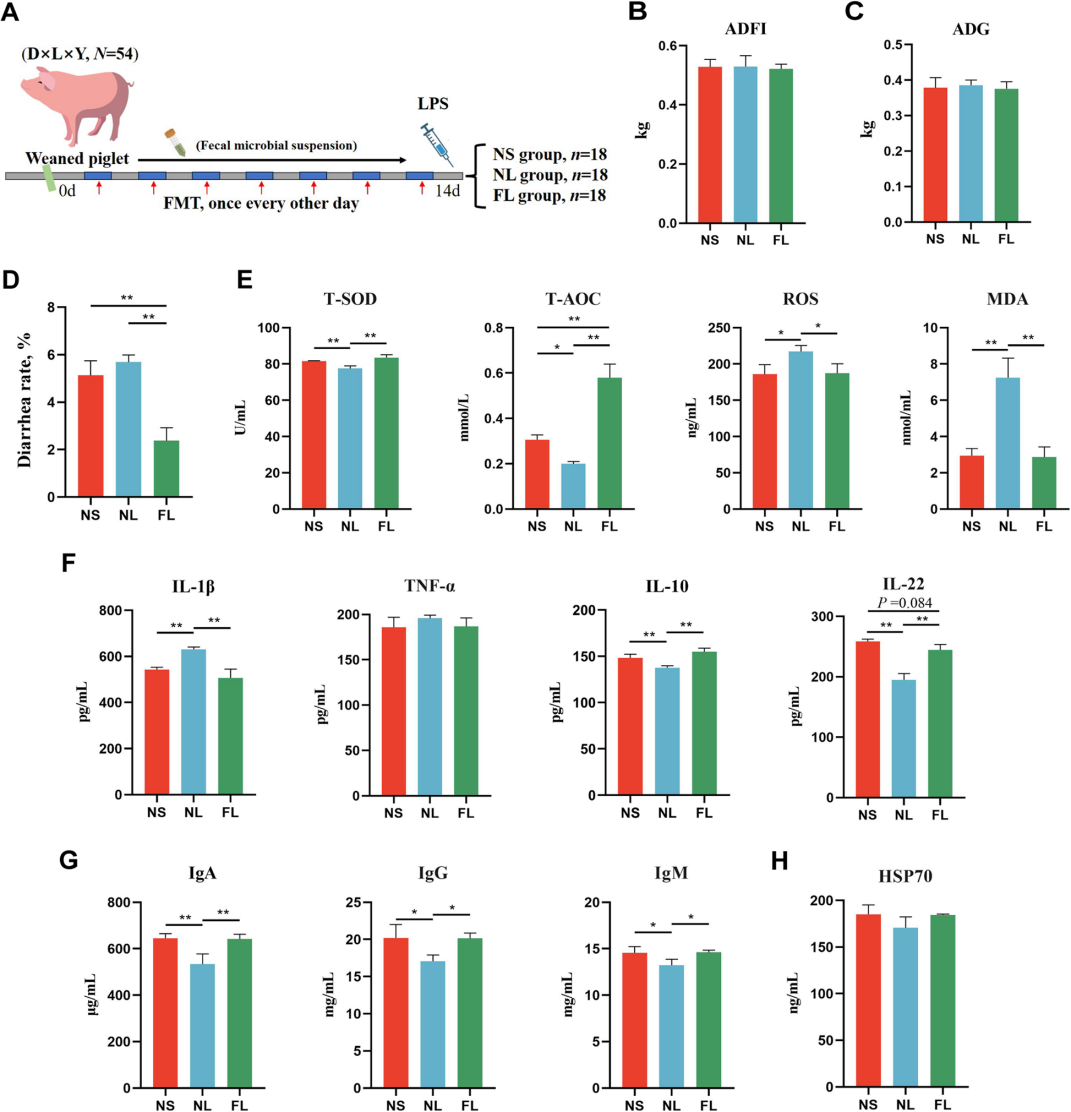
FMT alleviates oxidative stress in LPS-induced weaned piglets. **A** Schematic diagram of trial 2 model. **B** Average daily feed intake. **C** Average daily gain. **D** Diarrhea rate. **E** Effects of FMT on serum antioxidant capacity (T-SOD, T-AOC, ROS, and MDA) in LPS-induced piglets. **F** Effects of FMT on serum inflammatory cytokines (IL-1β, TNF-α, IL-10, and IL-22) in LPS-induced piglets. **G** Effects of FMT on serum immunoglobulins (IgA, IgG, and IgM) in LPS-induced piglets. **H** Effects of FMT on serum HSP70 levels in piglets. ^*^P < 0.05, ^**^P < 0.01, Data are shown as mean ± SD. *n* = 6

Subsequently, we established the LPS oxidative stress weaning piglet model to investigate the mechanism of diarrhea alleviation by FMT (Fig. 3A). Consistent with the previous results, FMT significantly alleviated diarrhea in weaned piglets at the end of the experiment (Fig. 3B–D). LPS could promote the production of ROS dramatically, which could lead to an imbalance of redox state in the host, cause oxidative stress, and result in oxidative damage to cells [31]. We found that LPS caused a significant decrease in T-SOD activity and T-AOC concentration, along with a significant increase in ROS and MDA levels, leading to oxidative stress in weaned piglets. In contrast, FMT treatment significantly alleviated oxidative stress in piglets and preserved the redox state (Fig. 3E). In addition, the oxidative stress caused by LPS led to severe inflammatory and abnormal immune responses in piglets. However, FMT reversed the LPS-induced significant increase in serum IL-1β and the significant decrease in IL-10 and IL-22 concentrations (Fig. 3F). Moreover, FMT treatment alleviated the LPS-induced significant reduction in serum immunoglobulin IgA, IgG, and IgM concentrations in weaned piglets, maintaining serum immunity levels (Fig. 3G–H).

### FMT alleviates LPS-induced intestinal injury in weaned piglets

**Fig. 4:**
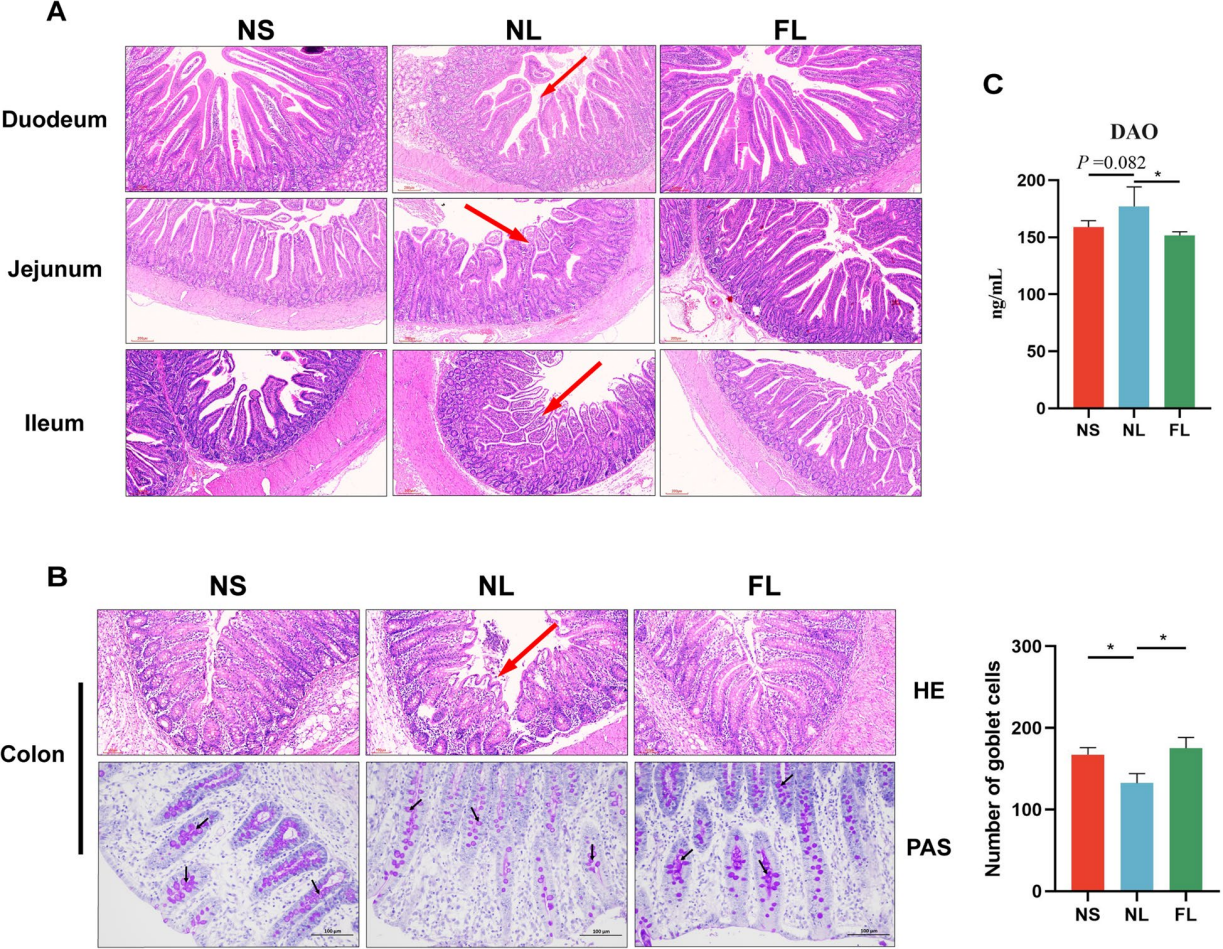
FMT alleviates intestinal injury in LPS-induced weaned piglets. **A** Effects of FMT on small intestinal morphology in LPS-induced piglets, with red arrows indicating intestinal villus damage. **B** Effects of FMT on colonic morphology (HE staining) and the number of goblet cells in colonic crypts (PAS staining) in LPS-induced piglets, with red arrows indicating colonic crypt damage and black arrows indicating goblet cells. **C** Effects of FMT on serum permeability marker DAO in LPS-induced piglets. ^*^P < 0.05, ^**^P < 0.01. Data are shown as mean ± SD. *n* = 6

**Fig. 5:**
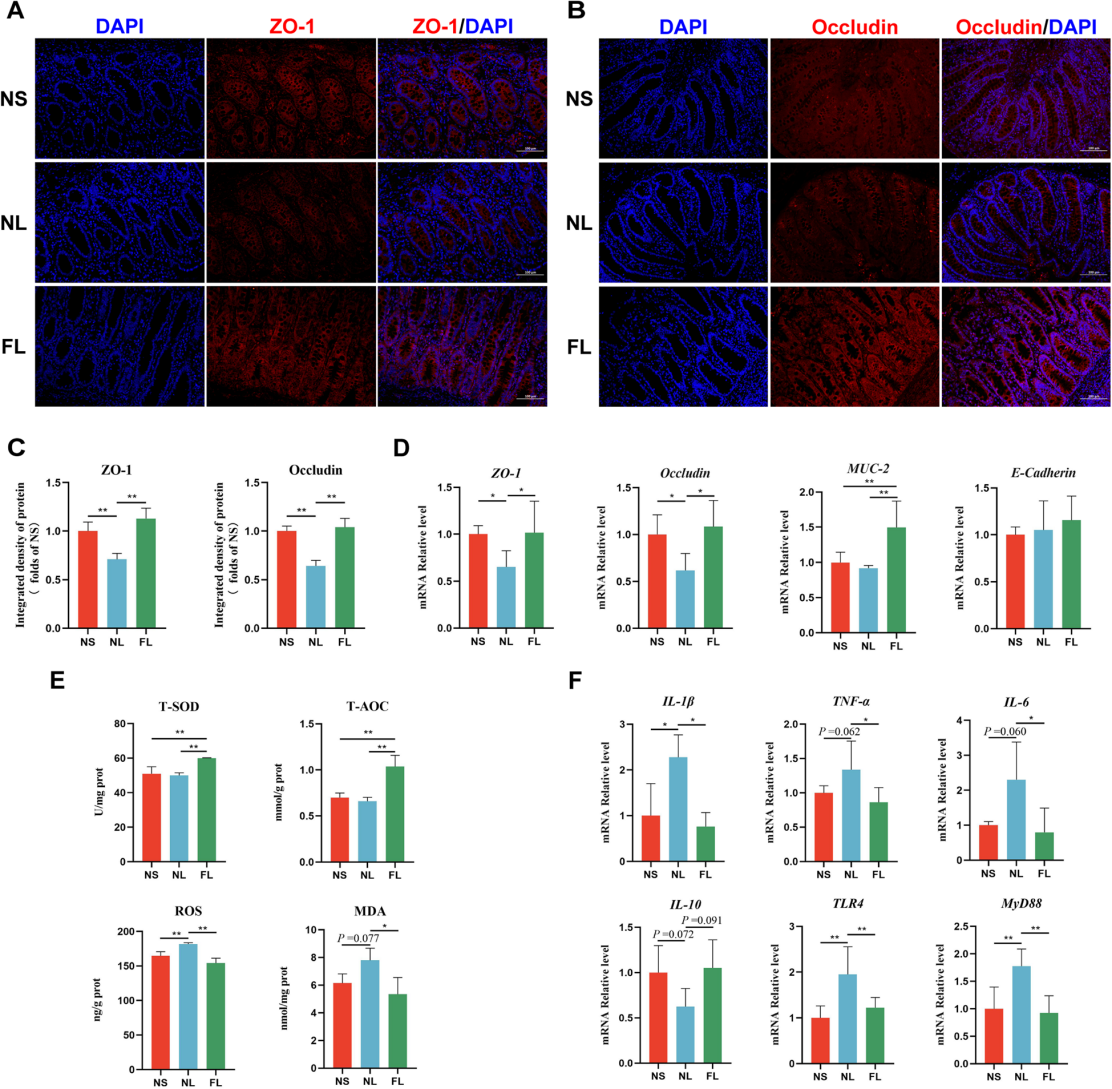
FMT improves colonic health in LPS-induced weaned piglets. **A**–**C** Representative immunofluorescence images of colonic barrier proteins ZO-1 and Occludin, along with their corresponding relative fluorescence intensity values. **D** Effects of FMT on the gene expression levels of colonic barrier proteins (*ZO-1*, *Occludin*, *MUC-2*, and *E-Cadherin*) in LPS-induced piglets. **E** Effects of FMT on the antioxidant capacity of colonic tissues (T-SOD, T-AOC, ROS, and MDA) in LPS-induced piglets. **F** Effects of FMT on colonic inflammation and NF-κB pathway factors (*IL-1β*, *TNF-α*, *IL-6*, *IL-10*, *TLR4*, and *MyD88*) in LPS-induced piglets. ^*^P < 0.05, ^**^P < 0.01. Data are shown as mean ± SD. *n* = 6

We performed HE staining of the intestines of weaned piglets and found that LPS caused severe breakage and destruction of villi in the duodenum, jejunum, and ileum. In contrast, FMT prevented LPS from destroying intestinal villi in the small intestine and maintained intestinal morphology (Fig. 4A). Subsequently, we observed the colonic morphology of piglets using HE and PAS staining. The results showed that LPS caused the breakage of the colonic crypts and significantly reduced the number of goblet cells in the crypts, which disrupted the morphology of the colon. However, the FMT treatment resulted in normal morphology of the colonic crypts, significantly increased the number of goblet cells in the crypts, and significantly decreased the serum DAO concentration (Fig. 4B and C). Colonic tissue immunofluorescence results showed that FMT significantly alleviated the LPS-induced reduction in the levels of colonic tight junction proteins ZO-1 and Occludin protein (Fig. 5A–C). Moreover, consistent with the histological observations, FMT significantly restored the LPS-induced decrease in gene expression of *ZO-1* and *Occludin* and significantly increased the gene expression of mucin *MUC-2* (Fig. 5D). Meanwhile, we found that FMT prevented LPS from causing significant increases in ROS and MDA concentrations in colon tissues, and significantly increased T-SOD activity and T-AOC concentration, thereby maintaining intestinal redox homeostasis (Fig. 5E). LPS promotes inflammatory cytokine expression by enhancing NF-κB signaling pathway expression [32]. We found that LPS significantly promoted *IL-1β*, *TNF-α*, and *IL-6* as well as *TLR4* and *MyD88* gene expression in colonic tissues, while FMT prevented intestinal inflammatory activation (Fig. 5F). These results indicated that FMT alleviates LPS-induced intestinal damage in weaned piglets and maintains intestinal morphology.

### FMT modulates LPS-induced colonic microbiota in weaned piglets to increase SCFA concentration

**Fig. 6:**
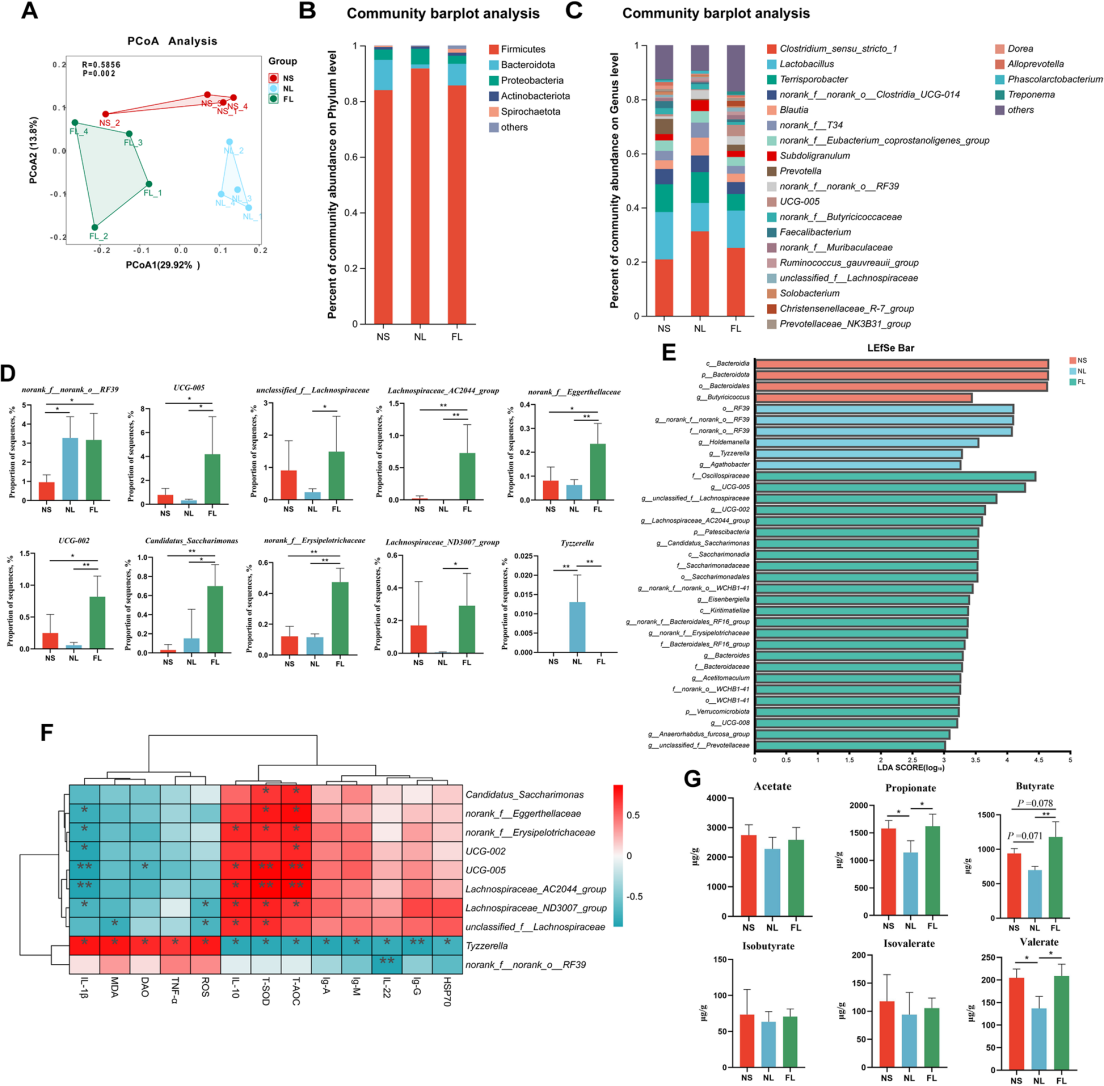
FMT modulates LPS-induced colonic microbiota in weaned piglets to increase SCFAs concentration. **A** Unweighted PCoA principal component analysis of the colonic microbiota in weaned piglets. **B** Phylum-level composition of the colonic microbiota in weaned piglets. **C** Genus-level composition of the colonic microbiota in weaned piglets. **D** Differential bacterial genera in the colon. **E** LEfSe analysis of the colonic microbiota in weaned piglets (LDA score ≥ 3). **F** Spearman correlation of serum immune and antioxidant capacity indices with differential colonic genera in weaned piglets. **G** Effects of FMT on colonic SCFA concentrations in LPS-induced weaned piglets. ^*^P < 0.05, ^**^P < 0.01. Data are shown as mean ± SD. *n* = 4

To further explore the impact of FMT on the gut microbiota of LPS-induced weaned piglets, we performed 16S rRNA gene sequencing for in-depth analysis of the colonic microbiota (Fig. S1A). Analysis of α-diversity indices revealed that FMT significantly increased the Sobs, Chao, and Ace indices of the colonic microbiota in LPS-induced piglets, enhancing the richness of the microbial community (Fig. S1B). PCoA analysis showed a clear separation and significant differences among the colonic microbiota samples of piglets from different treatment groups (Fig. 6A). Subsequently, we further investigated the differences in the colonic microbiota of piglets. At the phylum level, the dominant phyla in the colonic microbiota of piglets in the NS, NL, and FL groups were Firmicutes, Bacteroidetes, and Proteobacteria (Fig. 6B). At the genus level, the main genera in the colon of piglets in these groups were *Clostridium *sensu stricto* 1*, *Lactobacillus*, and *Terrisporobacter* (Fig. 6C). In addition, we found that FMT significantly increased LPS-induced colonization of piglets with *UCG-005*, *unclassified Lachnospiraceae*, *Lachnospiraceae AC2044 group*, *norank Eggerthellaceae*, *UCG-002*, *Candidatus Saccharimonas*, *norank Erysipelotrichaceae*, and *Lachnospiraceae ND3007 group* bacterial genera relative abundance, while significantly inhibiting the increase in the abundance of *Tyzzerella* genera (Fig. 6D). LEfSe analysis further showed that *UCG-005*, *unclassified Lachnospiraceae*, *UCG-002*, *Lachnospiraceae AC2044 group*, and *Candidatus Saccharimonas* could be used as marker differential genera for FMT (Fig. 6E). In addition, Spearman correlation analysis between differential genera and serum biochemical indices revealed that serum anti-inflammatory cytokines, immunoglobulins, and antioxidant capacity were positively correlated with *Candidatus Saccharimonas*, *norank Eggerthellaceae*, *norank Erysipelotrichaceae*, *UCG-002*, *UCG-005*, *Lachnospiraceae AC2044 group*, *Lachnospiraceae ND3007 group*, and *unclassified Lachnospiraceae*, and negatively correlated with *Tyzzerella* (Fig. 6F). As bacterial fermentation products in the intestine, SCFAs can act as signaling molecules to regulate the physiological functions of intestinal epithelial cells and improve the host health [33]. We measured colonic SCFA concentrations in piglets and found that FMT increased propionate, butyrate, and valerate levels in LPS-induced weaned piglets (Fig. 6G). This indicated that FMT regulated the structure of the gut microbial community, promoted the production of SCFAs, and improved intestinal health.

### FMT promotes LPS-induced riboflavin metabolism in weaned piglets.

**Fig. 7:**
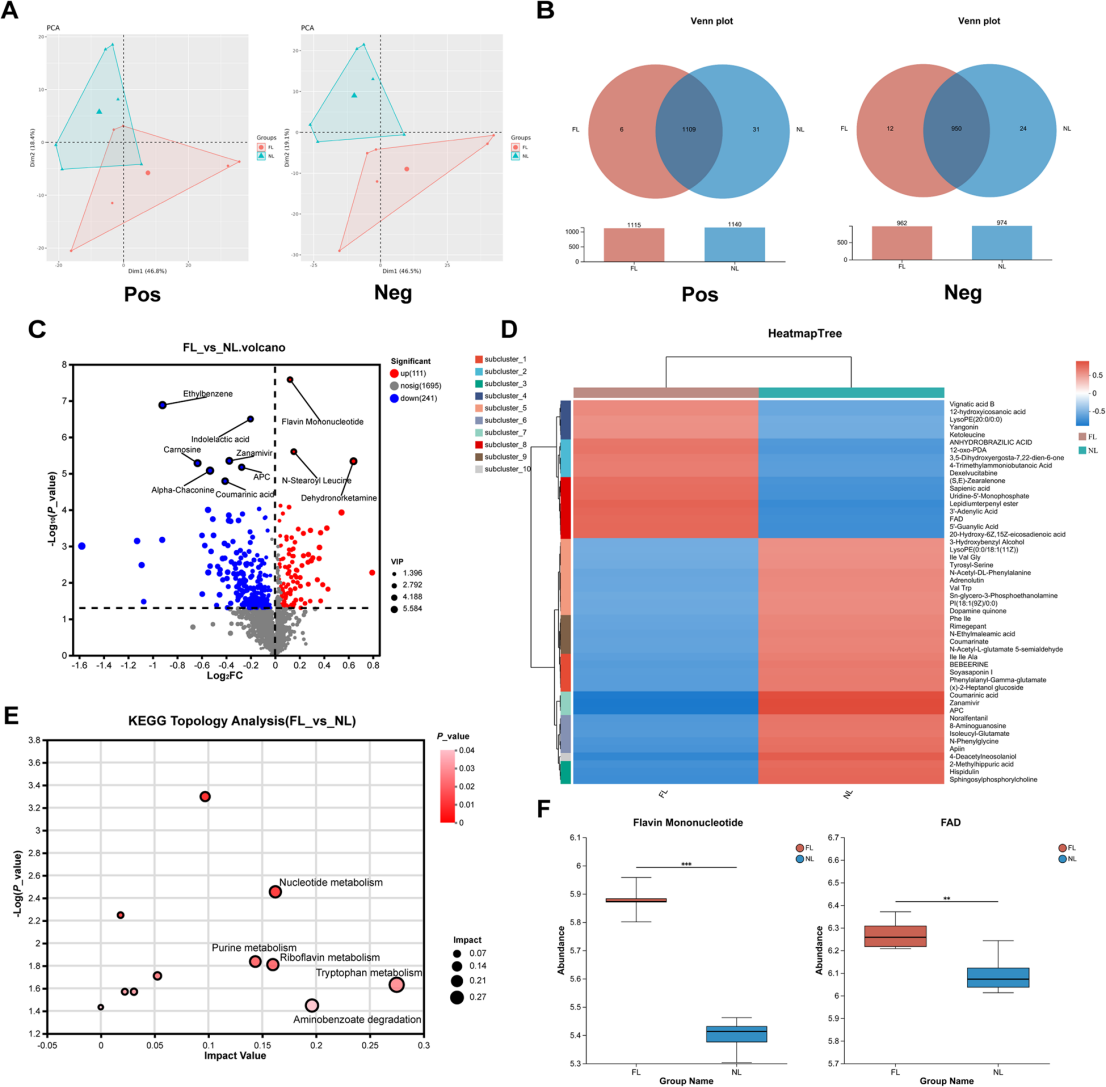
FMT promotes LPS-induced riboflavin metabolism in weaned piglets. **A** PCA of colonic metabolites (Pos: positive ion mode; Neg: negative ion mode). **B** Venn analysis of colonic metabolites (Pos: positive ion mode; Neg: negative ion mode). **C** Volcano plot of differential colonic metabolites, with red indicating significant upregulation and blue indicating significant downregulation. **D** Clustering heatmap of the top 50 differentially abundant colonic metabolites across treatment groups. **E** KEGG topological analysis of metabolic pathways of differential colonic metabolites from different treatment groups (significantly enriched pathways: *P* < 0.05). **F** Effects of FMT on the abundance of key metabolites in the riboflavin metabolism pathway (Flavin Mononucleotide and FAD) in LPS-induced weaned piglets. ^*^P < 0.05, ^**^P < 0.01, ^***^P < 0.001. Data are shown as mean ± SD. *n* = 6

Gut microbiota-derived metabolic products can directly influence physiological functions. We employed colonic untargeted metabolomics to investigate the effects of FMT on colonic metabolites in LPS-induced piglets. PCA and Venn analysis indicated that FMT pretreatment significantly altered colonic metabolites in LPS-induced piglets (Fig. 7A and B). Analysis of differential metabolites in the colon revealed that FMT upregulated 111 metabolites, including Flavin Mononucleotide (FMN), N-Stearoyl Leucine, and Dehydronorketamine, and downregulated 241 metabolites, including Ethylbenzene, Indolelactic acid, Zanamivir, Carnosine, APC, Alpha-Chaconine, and Coumarinic acid (Fig. 7C). Cluster analysis of the top 50 differentially abundant metabolites between the two groups revealed that, compared with the NL group, the relative abundances of Vignatic acid B, Yangonin, Ketoleucine, 12-oxo-PDA, Sapienic acid, Uridine-5'-Monophosphate, 3'-Adenylic Acid, FAD, and 5'-Guanylic Acid were higher in the FL group (Fig. 7D). Furthermore, KEGG topology analysis revealed that the differential metabolites were primarily involved in the top five significantly enriched metabolic pathways: Tryptophan metabolism, Aminobenzoate degradation, Riboflavin metabolism, Nucleotide metabolism, and Purine metabolism (Fig. 7E). Moreover, we found that FMT significantly increased the abundance of FMN and FAD, which are involved in the riboflavin metabolism pathway (Fig. 7F).

### FAD promotes GR activity, thereby enhancing antioxidant capacity.

**Fig. 8:**
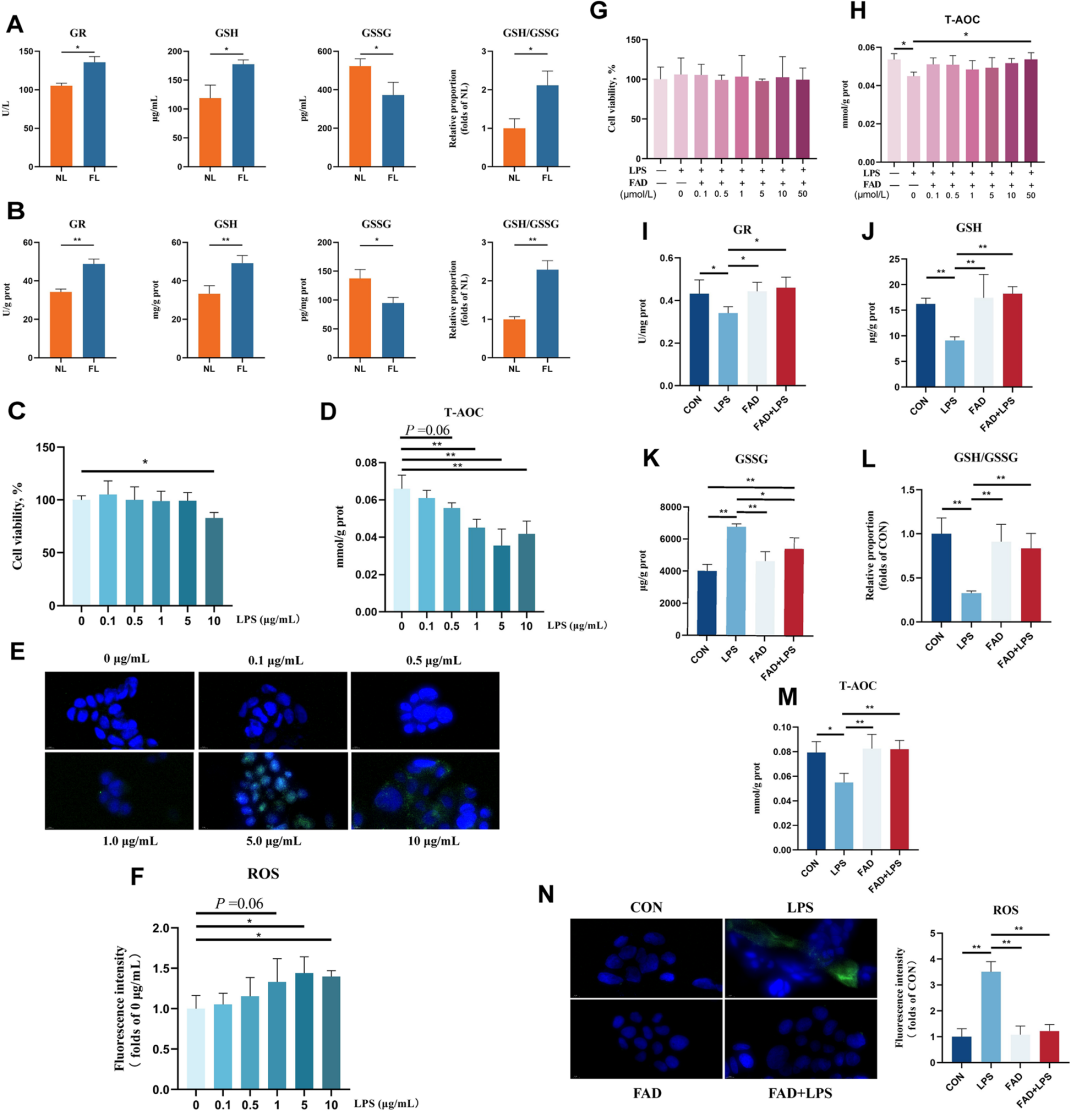
FAD promotes GR activity, thereby enhancing antioxidant capacity. **A** Effects of FMT on serum glutathione parameters (GR, GSH, GSSG, and GSH/GSSG) in LPS-induced weaned piglets. **B** Effects of FMT on colonic glutathione parameters (GR, GSH, GSSG, and GSH/GSSG) in LPS-induced weaned piglets. **C** and **D** Effects of different concentrations of LPS on Caco-2 cell viability and T-AOC levels. **E** and **F** Effects of different LPS concentrations on ROS levels in Caco-2 cells and the corresponding fluorescence quantification; green fluorescence indicates ROS levels. **G** and **H** Effects of different concentrations of FAD on cell viability and T-AOC levels in LPS-induced Caco-2 cells. **I**–**M** Effects of FAD on Caco-2 glutathione parameters (GR, GSH, GSSG, and GSH/GSSG) and T-AOC levels under LPS-induced conditions. **N** Effect of FAD on ROS levels in Caco-2 cells and the corresponding fluorescence quantification; green fluorescence indicates ROS levels. ^*^P < 0.05, ^**^P < 0.01. Data are shown as mean ± SD. *n* = 6

FAD can serve as a coenzyme for GR, facilitating the reduction of GSSG to GSH and maintaining the host's redox state [34]. Consistent with this, we found that FMT significantly increased GR activity, GSH levels, and the GSH/GSSG ratio in piglet serum and colonic tissue, while lowering GSSG levels (Fig. 8A and B). Additionally, we established an in vitro Caco-2 colon epithelial cell model to study the effect of FAD on antioxidant capacity in LPS-induced colon epithelial cells. Cell experiments with different concentrations of LPS and FAD revealed that 10 μg/mL LPS caused a significant drop in cell viability (Fig. 8C). Additionally, LPS at 1.0, 5.0, and 10 μg/mL significantly reduced cellular T-AOC levels, while 5.0 and 10 μg/mL LPS significantly increased cellular ROS levels (Fig. 8D–F). Therefore, we selected 5 μg/mL LPS to maintain normal cell viability and induce an oxidative stress model. Furthermore, we found that pretreatment of Caco-2 cells with 50 μmol/L FAD significantly alleviated the LPS-induced reduction in T-AOC levels (Fig. 8G and H). Therefore, 50 μmol/L FAD and 5 μg/mL LPS were used for the final cell experiment. Consistent with the in vivo results, FAD restored the LPS-induced reduction in GR activity, GSH levels, GSH/GSSG ratio, and T-AOC concentration while preventing the increase in GSSG levels and ROS accumulation (Fig. 8I–N). These results indicate that FMT can effectively enhance GR activity through FAD, improve antioxidant capacity, and maintain redox homeostasis in the body.

## Discussion

Due to significant physiological and environmental pressures, weaned piglets are susceptible to stress that disrupts the intestinal microbiota and elevates systemic inflammation, resulting in severe diarrhea and reduced growth performance, which further leads to slower subsequent growth and ultimately affects production efficiency [2]. In the past, antibiotics were commonly incorporated into pig diets to prevent diarrhea in piglets [35]. However, given the threats that antibiotics pose to global ecosystems and food safety, the livestock industry is urgently seeking novel antibiotic replacement additives [36]. It has been reported that the addition of various types of dietary fiber to weaned piglet diets can effectively modulate the gut microbiota, improve intestinal health, and enhance growth performance [37, 38]. Previous research by Sun et al. indicated that alfalfa fiber might improve growth performance by modulating the structure of the intestinal microbiota in weaned piglets [20]. In fact, the intestinal microbiota and its metabolic products play a crucial role in maintaining intestinal barrier function and overall health [39]. In recent years, various studies have indicated that early administration of fecal microbial suspensions from different sources to piglets can achieve superior outcomes [24–26]. Therefore, we further prepared fecal microbial suspensions from piglets fed alfalfa fiber meal diet and performed FMT on weaned piglets. The results were consistent with previous reports [28], demonstrating that FMT significantly reduced inflammatory levels in weaned piglets and enhanced both immune and antioxidant capacities. Moreover, intestinal morphological development directly affects the efficiency of nutrient utilization in piglets [40]. We found that FMT significantly improved the morphology of the jejunum and the development of colonic crypts in piglets, and effectively prevented the leakage of DAO into the bloodstream. Fecal microbiota, primarily originating from the donor colon, may have a more pronounced effect in the recipient's colon [41, 42]. We further investigated the colonic barrier function in piglets and found that FMT increased levels of colonic tight junction proteins and expression of the mucin *MUC-2* gene, thereby enhancing intestinal barrier function and reducing the risk of intestinal infection and inflammation. These results indicate that FMT can promote the healthy development of weaned piglets by modulating the gut microbiota.

To further investigate the effects and underlying mechanisms of the fecal microbiota on weaning stress in piglets, we established an oxidative stress model in weaned piglets using LPS induction. LPS stimulates the body to produce large amounts of ROS, resulting in an imbalance in the redox state, which triggers oxidative stress, disrupts antioxidant capacity, and elicits severe inflammatory responses [43, 44]. We found that FMT alleviated LPS-induced increases in ROS and the lipid peroxidation product MDA in the serum and intestinal tissues of weaned piglets, while enhancing T-SOD and T-AOC levels to maintain normal redox balance. Moreover, FMT effectively countered the LPS-induced rupture and damage of small intestinal villi and colonic crypts, and prevented DAO from leaking into the bloodstream. It has been reported that LPS can activate the NF-κB signaling pathway, thereby promoting the expression of inflammatory cytokines and leading to cell damage and death [32]. We found that FMT pretreatment significantly prevented the LPS-induced increase in the expression of *TLR4* and *MyD88* genes in the NF-κB signaling pathway in the colon of weaned piglets, as well as the elevation of inflammatory cytokines, thereby improving immune status. These findings indicate that fecal microbiota can mitigate LPS-induced oxidative stress in weaned piglets and help maintain overall health.

Intestinal microbiota can influence various physiological characteristics of the host animal, including growth and development, nutrient metabolism, and immune function [45]. However, various external stimuli can disrupt the gut microbiota, impair intestinal barrier function and the immune system, and increase the risk of pathogen invasion and infection [46]. Many studies have shown that early FMT in weaned piglets can improve the composition of the gut microbiota, regulate intestinal immunity and barrier function, and enhance overall health [23–25]. This study found that FMT increased the richness of the colonic gut microbiota in LPS-induced weaned piglets and significantly altered the composition of microbial species. FMT increased the relative abundances of bacterial genera in the colon of LPS-induced weaned piglets, including *UCG-005*, *Lachnospiraceae AC2044 group*, *norank Eggerthellaceae*, *UCG-002*, *Candidatus Saccharimonas*, *norank Erysipelotrichaceae*, *unclassified Lachnospiraceae*, and *Lachnospiraceae ND3007 group*. According to reports, most of these genera are associated with SCFA production and exert beneficial effects on intestinal health and immunity [47–49]. In addition, FMT pretreatment significantly suppressed the relative abundance of the *Tyzzerella* genus in the colon of LPS-induced weaned piglets. Studies have shown that, compared with healthy populations, the abundance of the *Tyzzerella* genus is significantly elevated in patients with enteritis, and it may have deleterious effects on cardiovascular and other diseases [50]. SCFAs, as important metabolic products of gut bacteria, can serve as an energy source for animals and act as cellular signaling molecules to regulate the physiological functions of intestinal epithelial cells, exerting anti-inflammatory and various beneficial effects [51, 52]. In this study, FMT significantly increased the concentrations of propionate, butyrate, and valerate in the colon of LPS-induced weaned piglets, particularly butyrate. Butyrate plays a crucial role in maintaining mucosal barrier function and immune regulation [11]. Yang et al. reported that butyrate derived from the gut microbiota can promote immune cells to produce IL-22, thereby exerting immunoregulatory effects [12]. Additionally, butyrate can mitigate intestinal barrier damage in weaned piglets induced by vomitoxins by promoting mitochondrial homeostasis [53]. Our findings are consistent with previous studies, suggesting that butyrate may inhibit the LPS-induced activation of the TLR4/NF-κB pathway, thereby exerting anti-inflammatory effects [54]. Our results indicate that FMT can regulate the gut microbiota composition in the colon of piglets, enhance microbial homeostasis, promote the colonization of beneficial bacteria while inhibiting harmful bacteria, increase intestinal SCFA concentrations, and strengthen intestinal barrier and immune functions, thereby improving overall health.

Metabolites derived from the gut microbiota can directly participate in host metabolic activities and play crucial physiological regulatory roles. We found that FMT significantly altered the gut metabolite profile in LPS-induced piglets and notably increased the relative abundance of FMN and FAD in the riboflavin metabolism pathway. Additionally, KEGG topological analysis indicated that riboflavin metabolism is one of the key metabolic pathways affected by differential metabolites. Riboflavin deficiency can impair the body's redox state and contribute to various diseases, including neurological disorders and cancer [55]. Several studies have shown that dietary supplementation with riboflavin in weaned piglets can significantly enhance antioxidant capacity, improve intestinal morphology, and promote the proliferation and differentiation of intestinal epithelial cells [56, 57]. Furthermore, research has demonstrated that riboflavin can reduce the mortality of mice suffering from LPS-induced septic shock by lowering pro-inflammatory cytokine levels [58]. In fact, FMN and FAD are the main bioactive forms of riboflavin, serving as essential cofactors for various oxidases, reductases, and dehydrogenases involved in redox processes. They play a crucial role in energy metabolism within the body [59]. FAD is a key component of the intracellular multifunctional oxidase system, primarily exerting its antioxidant function by acting as a coenzyme for GR, facilitating the conversion of GSSG to GSH to maintain intracellular glutathione levels [34, 60]. GSH is a crucial antioxidant in the body, playing a key role in defending against ROS-induced oxidative stress and maintaining normal redox balance [61]. In this study, we found that FMT pretreatment significantly reduced ROS concentrations in serum and tissues while increasing GR activity and GSH levels. This suggests that FAD, a key metabolite in the riboflavin metabolism pathway, may play a critical role in FMT-mediated alleviation of LPS-induced oxidative stress in piglets. Subsequently, we used LPS to induce oxidative stress in Caco-2 cells in vitro to simulate the oxidative stress model. The experimental results were consistent with in vivo findings, showing that FAD treatment significantly increased GSH, GR, and T-AOC levels, as well as the GSH/GSSG ratio, while markedly reducing GSSG and ROS levels in LPS-induced cells. This indicates that FMT alleviates LPS-induced oxidative stress in piglets through FAD acting as a GR coenzyme, helping to maintain redox homeostasis and improve overall health. Therefore, our findings underscore the potential of FMT from specifically selected donors. As research on targeted FMT advances, this approach holds promise for mitigating stress-induced impairments during critical developmental windows in young animals and human infants, offering a novel pathway for microecological intervention.

## Conclusion

**Fig. 9:**
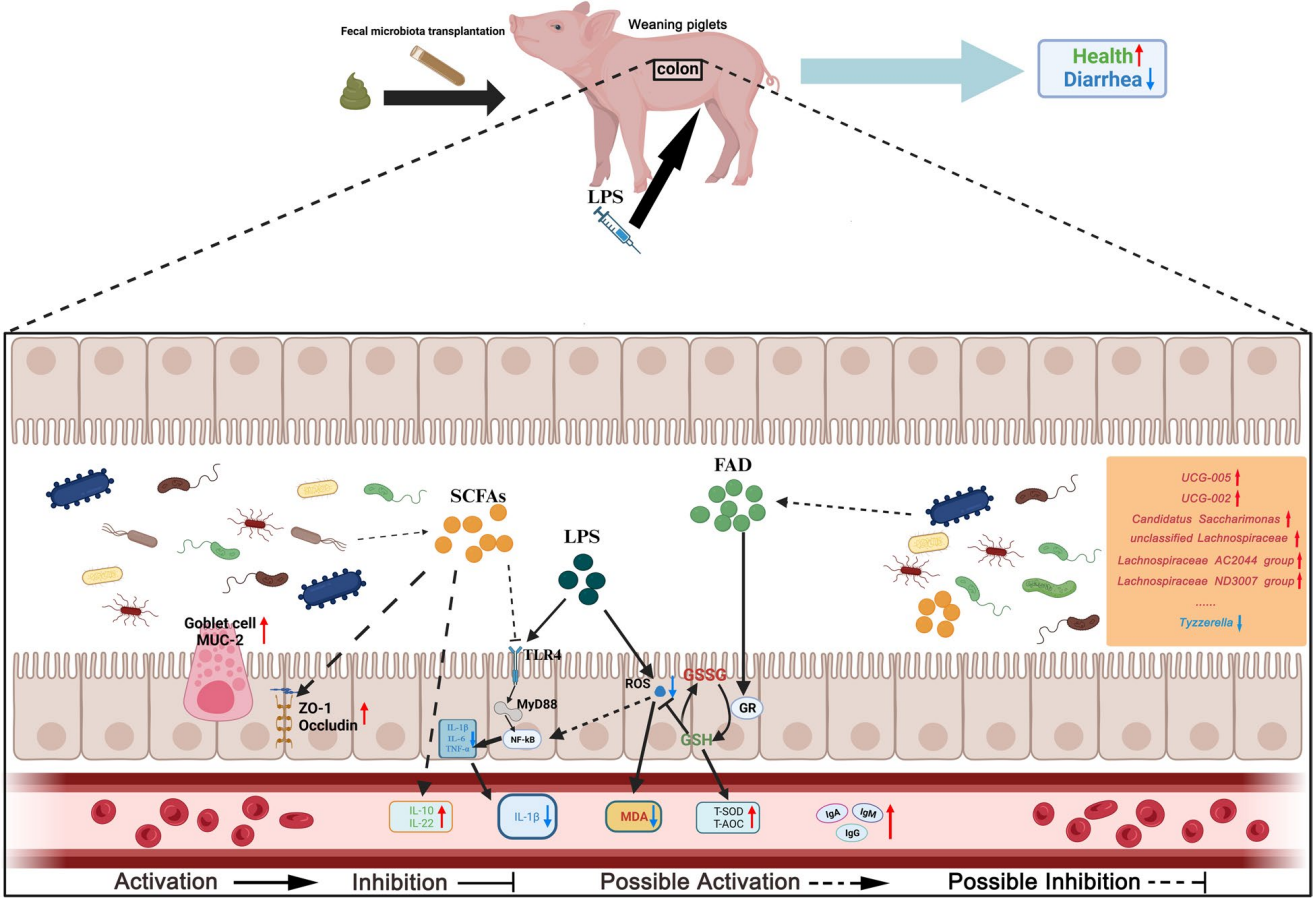
FMT from donor pigs fed an alfalfa fiber-based diet modulates the gut microbiota, increases intestinal SCFA and FAD levels, enhances intestinal immune and antioxidant capacities, and alleviates diarrhea in weaned piglets. Fecal microbiota transplantation from donor piglets whose diets are supplemented with alfalfa fiber can improve the colonic microbial community structure in LPS-induced oxidative stress in weaned piglets, increase colonic SCFA and FAD levels, enhance GR activity, maintain GSH levels, prevent LPS-induced elevation of ROS and activation of NF-κB, lower pro-inflammatory cytokine levels, and improve intestinal immune, antioxidant, and barrier functions, thereby promoting overall health and alleviating diarrhea

In conclusion, our results indicated that the fecal microbiota from piglets fed an alfalfa fiber-supplemented diet helps mitigate early weaning diarrhea and oxidative stress induced by LPS in weaned piglets. This effect is achieved by modulating the gut microbiota composition, increasing intestinal SCFA and FAD levels, and enhancing gut immunity and antioxidant capacity (Fig. 9).

## Supplementary Information

Additional file 1: Table S1. The fecal donor information. Table S2. Primer sequences of genes. Fig. S1. (A) Rarefaction curves of microbiota. (B) Alpha diversity analysis of microbiota.

## Data Availability

Data will be made available on request.

## Abbreviations

ADG: Average daily gain
ADFI: Average daily feed intake
CD: Crypt depth
DAO: Diamine oxidase
FAD: Flavin adenine dinucleotide
FMN: Flavin mononucleotide
FMT: Fecal microbiota transplantation
GAPDH: Glyceraldehyde-3-phosphate dehydrogenase
GR: Glutathione reductase
GSH: Reduced glutathione
GSSG: Oxidized glutathione
HSP 70: Heat shock protein 70
IgA: Immunoglobulin A
IgG: Immunoglobulin G
IgM: Immunoglobulin M
IL-1β: Interleukin-1β
IL-10: Interleukin-10
IL-22: Interleukin-22
LPS: Lipopolysaccharide
MDA: Malondialdehyde
MUC-2: Mucin 2
MyD88: Myeloid differentiation primary response gene 88
NF-κB: Nuclear factor kappa-light-chain-enhancer of activated B cells
ROS: Reactive oxygen species
SCFAs: Short-chain fatty acids
T-AOC: Total antioxidant capacity
T-SOD: Total superoxide dismutase
TLR4: Toll-like receptor 4
TNF-α: Tumor necrosis factor-α
VH: Villus height
VCR: Villus-to-crypt ratio
ZO-1: Zonula Occludens-1
