# Supplementary material for: Fecal microbiota transplantation mitigates lipopolysaccharide-induced oxidative stress in weaned piglets by modulating gut microbiota and enhancing riboflavin metabolism
Source: J Anim Sci Biotechnol. 2026 Jan 16;17:9. doi: 10.1186/s40104-025-01330-6 (PMC12809982; doi:10.1186/s40104-025-01330-6)
Supplement: Supplementary file 1 — Additional file 1: Table S1. The fecal donor information. Table S2. Primer sequences of genes. Fig. S1. (A) Rarefaction curves of microbiota. (B) Alpha diversity analysis of microbiota. [file 40104_2025_1330_MOESM1_ESM.docx]

**Table S1 The fecal donor information.**

| Donor | Gender | Age/days | Body weight/kg |
| --- | --- | --- | --- |
| Donor 1 | male | 60 | 25.70 |
| Donor 2 | male | 60 | 25.45 |
| Donor 3 | male | 60 | 25.37 |
| Donor 4 | male | 60 | 25.12 |
| Donor 5 | male | 60 | 26.03 |
| Donor 6 | male | 60 | 25.60 |

**Table S2 Primer sequences of genes.**

| Genes | Primer sequence (5'—3') | Accession number |
| --- | --- | --- |
| *ZO-1* | F: GAGGCTCAGCCCTATCCATCTG  R: CGGGACCTGCTCATAACTTCGT | XM_005659811.1 |
| *Occludin* | F: CGGCCATATCCAGAGTCTTCGT  R: CGTTTTGAAGACGCCTCCAAG | NM_001163647.2 |
| *E-Cadherin* | F: CCCCAACACTTCTCCCTTCACT  R: CTCGAGGGTTTTCTTTGGCTTC | EU805482.1 |
| *MUC-2* | F: ACACCATCTACCTCACTCAGC  R: TCCTCTCTGTTCCACACGA | XM_013989745.1 |
| *IL-1β* | F: TCCTCACAGGGGACTTGA  R: GGGTGGGCGTGTTATCT | XM_021085847.1 |
| *IL-6* | F: CTTCTGGTGATGGCTACTG  R: GATTTTGCCGAGGATGTA | NM_214399.1 |
| *TNF-α* | F: CACCACGCTCTTCTGCCTACT  R: CGGCTTTGACATTGGCTACA | NM_214022.1 |
| *IL-10* | F: CTTGTCAGAGATGATCCAGTTTT  R: TTCACCTCCTCCACGGC | NM_214041.1 |
| *TLR4* | F: TGCCCCTACTCAATCTCTCT  R: CAAGTTTTCATTACATCCGAAC | NM_001030693.1 |
| *MyD88* | F: TGGAACAGACCAACTATCGGC  R: CATCAGAGACAACCACTACCATCC | NM_001099923.1 |
| *GAPDH* | F: CGTCCCTGAGACACGATGGT  R: GCCTTGACTGTGCCGTGGAAT | NM_001206359.1 |


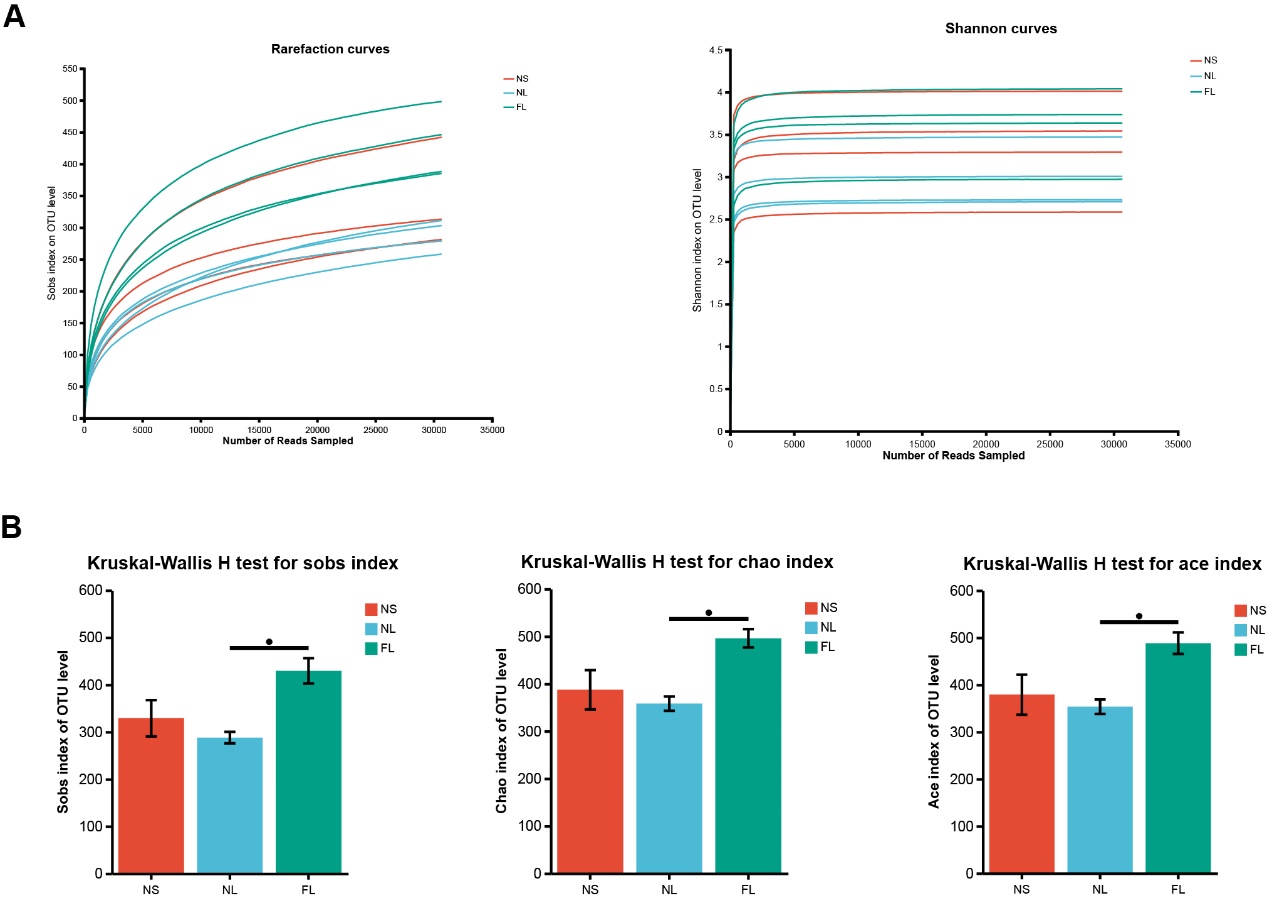


**Fig. S1 (A) Rarefaction curves of microbiota. (B) Alpha diversity analysis of microbiota.**
